# An author’s reply to the editorial “Torque Teno virus load as a surrogate marker for the net state of immunosuppression: The beneficial side of the virome”

**DOI:** 10.1111/ajt.15962

**Published:** 2020-05-10

**Authors:** Konstantin Doberer, Gregor Bond

**Affiliations:** ^1^ Division of Nephrology and Dialysis Department of Medicine III Medical University Vienna Austria

**Keywords:** complication: infectious, editorial/personal viewpoint, immunosuppressant, immunosuppression/immune modulation, infection and infectious agents – viral, infectious disease, kidney transplantation/nephrology, monitoring: immune, rejection

*To the Editor:*

We are grateful for the editorial by Fernández‐Ruiz,^1^ highlighting the value of Torque Teno virus (TTV) quantification for risk stratification of graft rejection and infection after kidney transplantation. However, we would like to correct some details concerning timing of TTV quantification and biopsies in our trial, comment on implications of the trial results for TTV replication control, and give an update on TTV standardization.

It is crucial to point out that the suggested TTV range for optimal immunosuppression in our trial is not restricted to month 3 post–kidney transplantation, as described by the editorial, but covers months 3‐12, providing potential guidance for immunosuppression during this period. The median time of TTV assessment for the association between TTV and rejection was 154 days and for the association between TTV and infection 180 days post–kidney transplantation, respectively. The editorial also mentions that the trial was restricted to patients who underwent for‐cause biopsy prior to TTV quantification. In fact, the analysis on the association between TTV level and graft rejection was restricted to TTV with subsequent for‐cause biopsy.

The editorial claims that our data support TTV replication control being mainly exerted by T–cell‐mediated immunity. We agree that T–cell immunity is considered to be crucial for TTV control. However, the literature also suggests immune compartments other than T cells to play a role: Implication of an antibody response to TTV was provided by Maggi and Bendinelli;^2^ Giacconi et al^3^ found evidence for a contribution of natural killer cells for TTV control and Rochi et al^4^ provided evidence for antibody presenting cells and toll‐like receptor TTV antigen recognition, which, of course, is indispensable for any T cell response and does not necessarily implicate innate immunity to be involved in TTV control independently of T cell action. Beyond experimental findings, however, work from our group has shown that TTV load inversely associates with antibody‐mediated rejection in kidney transplant patients.^5^ Main effectors of antibody‐mediated rejection are currently considered to be natural killer cells, the complement system, and donor‐specific antibodies. In this respect, our finding provides evidence for an association of TTV load and the function of these immune compartments. In addition, we have shown that TTV load increases in patients with rheumatoid arthritis after application of anti B cell agents.^6^ These data provide evidence for TTV levels mirroring B cell function.

Finally, the editorial mentioned the lack of international standards for TTV polymerase chain reaction (PCR) assays. In this respect, it is important to note that such standardization process has been initiated in 2018 by an External Quality Assessment pilot study by Quality Control for Molecular Diagnostics. First preliminary data were presented at the 22nd Annual Meeting of the European Society for Clinical Virology: accuracy in TTV quantification was shown in all participating laboratories.^7^

Taken together, we provide crucial corrections concerning the design of our trial, a short overview of immune compartments involved in TTV replication control, and an update on TTV PCR standardization in this letter to the editor.

## DISCLOSURE

The authors of this manuscript have no conflicts of interest to disclose as described by the *American Journal of Transplantation.*
